# Preliminary Insights into the Gut Microbiota of Captive Tigers in Republic of Korea: Influence of Geographic and Individual Variation

**DOI:** 10.3390/microorganisms13061427

**Published:** 2025-06-19

**Authors:** Beoul Kim, Saebom Lee, You-Jeong Lee, Yong-Myung Kang, Man Hee Rhee, Dongmi Kwak, Yong-Gu Yeo, Ju Won Kang, Taehwan Kim, Min-Goo Seo

**Affiliations:** 1 College of Veterinary Medicine & Institute for Veterinary Biomedical Science, Kyungpook National University, 80 Daehak-ro, Buk-gu, Daegu 41566, Republic of Korea; kbjjhnm@naver.com (B.K.); wowgirlsgood@naver.com (Y.-J.L.); kamaboy88@knu.ac.kr (Y.-M.K.); rheemh@knu.ac.kr (M.H.R.); dmkwak@knu.ac.kr (D.K.); 2 Baekdudaegan National Arboretum, Korean Tiger Coservation Center, 1501 Chunyang-ro, Chunyang-myeon, Bonghwa 36209, Republic of Korea; gltp43@koagi.or.kr; 3 Conservation and Health Center, Seoul Zoo, 102 Daegongwongwangjang-ro, Gwacheon-si 13829, Republic of Korea; withyonggu@seoul.go.kr; 4 Veterinary Medicine Team, Uchi Park Zoo, 677, Uchi-ro, Buk-gu, Gwangju 61028, Republic of Korea; gjw0210@korea.kr

**Keywords:** tiger gut microbiome, microbial diversity, 16S rRNA gene sequencing, Siberian tiger, Bengal tiger, captivity, conservation

## Abstract

The gut microbiome plays a crucial role in the health and physiology of tigers (*Panthera tigris*), influencing digestion, immune function, and overall well-being. While numerous studies have characterized the gut microbiota of domestic carnivores and some wild felids, comparative analyses across different tiger subspecies under varying environmental contexts remain limited. In this exploratory study, we investigated the gut microbiome diversity and composition of 15 captive tigers, including both Siberian (*P. tigris altaica*) and Bengal (*P. tigris tigris*) subspecies, housed in two different regions in Korea. Using 16S rRNA gene sequencing of fecal samples, we analyzed microbial diversity across multiple taxonomic levels. Preliminary analyses revealed significant differences in microbial composition between geographic locations, whereas sex-based differences appeared minimal. Alpha and beta diversity metrics demonstrated substantial inter-individual variability, likely influenced by regional and environmental factors. Given the small sample size and the confounding between subspecies and housing location, the findings should be regarded as preliminary and not generalized beyond this specific cohort. Nevertheless, these insights highlight the potential utility of gut microbiome profiling for health monitoring and management in captive-tiger populations. Future research incorporating larger, more diverse cohorts will be essential to validate these trends and clarify the roles of diet, health status, and enrichment in shaping the gut microbiota.

## 1. Introduction

The gut microbiome is essential to host health, influencing digestion, immune function, and metabolic regulation. Recent research highlights the importance of microbial diversity in wildlife species, particularly in large carnivores, where microbiome composition is influenced by diet, genetics, environmental exposure, and captivity [1]. In captive settings, multiple environmental factors—including enclosure size and substrate, sanitation and hygiene protocols, veterinary interventions (e.g., antibiotic use), and psychological stress due to limited stimuli—can collectively alter the gut microbial environment. These factors may contribute to microbial dysbiosis, immune suppression, and increased disease susceptibility in long-term captive individuals [2,3].

Tigers (*Panthera tigris*), classified as endangered by the International Union for Conservation of Nature, face significant conservation challenges due to habitat loss, poaching, and declining genetic diversity [4]. Although microbiome studies conducted on domestic carnivores, such as dogs and cats [5,6] are extensive, there is a growing body of literature on wild felids, including lions, and cheetahs. For instance, studies on African lions (*Panthera leo*) have demonstrated that changes in gut microbial composition are associated with habitat encroachment and dietary shifts [7]. Similarly, cheetahs (*Acinonyx jubatus*) in captivity have shown decreased microbial diversity and increased susceptibility to gastrointestinal disorders [8]. These findings provide important comparative insights for evaluating the tiger microbiome. A study shows that captivity influences microbial diversity by altering dietary composition, movement patterns, and environmental microbiota exposure [9]. Studies on wild tigers in India and Russia report greater microbial richness, potentially owing to a more diverse natural diet and environmental exposure, compared to captive tigers fed standardized diets [10]. Captive tigers may also experience gut microbiome shifts associated with health conditions, including metabolic disorders, gastrointestinal issues, and chronic inflammation [11].

Mechanistically, gut microbes interact with the host through multiple pathways, such as the fermentation of dietary fibers into short-chain fatty acids (SCFAs), the modulation of systemic immunity, and the maintenance of intestinal-barrier integrity [12]. Disruption in microbial diversity has been linked to increased intestinal permeability, systemic inflammation, and reduced metabolic efficiency—all of which may have significant consequences for endangered species under captive care [1]. The gut microbiomes of six captive Siberian tigers were analyzed in a previous study in Korea, providing initial insights into microbial composition in this population [13]. The study reports that the individual’s health status, diet, and environmental factors influence gut microbial diversity. However, the study was limited by a small sample size and the absence of comparative analyses across different subspecies or geographic locations.

Based on these findings, this exploratory study investigates the gut microbiomes of 15 captive tigers in Korea, including Siberian (*P. tigris altaica*) and Bengal tigers (*P. tigris tigris*), to evaluate whether microbial diversity varies by subspecies, sex, or regional differences. The gut-microbiome composition at multiple taxonomic levels was characterized, and the alpha and beta diversity indices were examined to assess microbial richness, evenness, and compositional differences. The potential influence of sex and geographic location was evaluated through comparative statistical analyses, with findings compared to those of previous studies on both wild and captive tigers. While this study offers preliminary insights that may inform microbiome-based health monitoring and welfare strategies in captive tiger populations, key limitations must be acknowledged. Notably, subspecies (Siberian vs. Bengal) and housing region (Seoul vs. Gwangju) were confounded in the sampling design, making it impossible to disentangle their individual effects. Therefore, the findings should not be generalized beyond the studied cohort but rather serve as a foundational step for future research involving larger and more diverse populations.

## 2. Results

### 2.1. Alpha Diversity Outcomes and Interpretations

**Figure 1:**
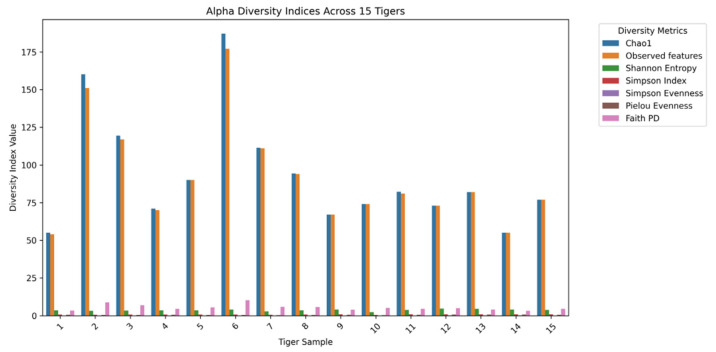
Bar plots illustrating alpha diversity indices (Chao1, Observed Features, Shannon Entropy, the Simpson Index, Simpson Evenness, Pielou Evenness, and Faith PD) for the gut microbiomes in 15 tigers.

Alpha diversity indices provided insights into microbial richness, evenness, and phylogenetic diversity within the gut microbiomes of 15 tigers. Microbial diversity was assessed using key indices, including Chao1, Observed Features, Shannon Entropy, the Simpson Index, Simpson Evenness, Pielou Evenness, and Faith’s Phylogenetic Diversity (Faith PD) (Figure 1). These metrics offered a comprehensive understanding of the microbial composition within each sample.

Chao1 and Observed Features are indicators of species richness, with higher values suggesting greater microbial diversity. Tigers 6 and 2 exhibited the highest richness, while Tigers 1 and 14 showed the lowest. Shannon Entropy and the Simpson Index measured both richness and evenness. Tigers 12, 13, and 14 demonstrated the highest diversity, whereas Tiger 10 had the lowest Shannon index, indicating reduced evenness. Simpson and Pielou Evenness indicate the uniformity of species distribution. Tigers 12 and 14 exhibited the highest evenness, while Tigers 2 and 7 had the lowest, suggesting dominance by fewer taxa. Faith PD represents the evolutionary diversity within the microbial community. Tigers 6 and 2 displayed the highest phylogenetic diversity, whereas Tigers 14 and 1 had the lowest.

**Figure 2:**
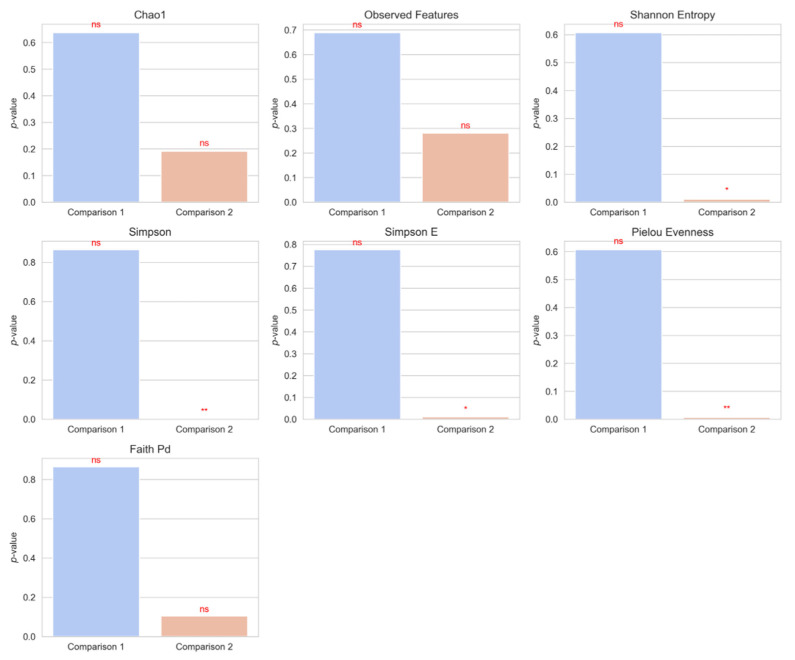
Statistical comparisons of alpha diversity indices between groups (Comparison 1: female vs. male, Comparison 2: Gwangju vs. Seoul) using the Wilcoxon rank-sum test. Statistical significance is indicated as follows: *p* < 0.01 as “**”, *p* < 0.05 as “*”, and not significant (ns) for *p* ≥ 0.05.

To determine significant differences in alpha diversity between groups, statistical comparisons were performed, focusing on two primary comparisons (Figure 2). In our analysis, alpha diversity indices were compared between female and male tigers (Comparison 1), revealing no statistically significant differences (*p* > 0.05). This suggests that gender does not substantially influence microbial richness and evenness. Conversely, when comparing tigers from Gwangju and Seoul (Comparison 2), significant differences were observed in Shannon Entropy (*p* = 0.0103), Simpson Evenness (*p* = 0.00293), and Pielou Evenness (*p* = 0.00586). These findings suggest that tigers from Seoul may possess a more evenly distributed and diverse microbial community compared to those from Gwangju, although caution is warranted due to the small sample size.

Alpha diversity analysis revealed that microbial richness remained stable across gender groups but varied significantly between different geographical locations. The observed differences in evenness-related indices (Shannon Entropy, Simpson Evenness, and Pielou Evenness) in Comparison 2 highlight the potential role of environmental or dietary factors in shaping gut-microbiome diversity. This finding provides preliminary insight into how regional variation may contribute to microbiome composition differences in captive tiger populations, though further validation with larger samples is needed.

### 2.2. Beta Diversity Outcomes and Interpretations

Beta diversity analysis was conducted to evaluate differences in microbial community composition among the 15 tigers. We employed multiple distance metrics, including Bray–Curtis, Jaccard, Unweighted UniFrac, and Weighted UniFrac, to assess microbial dissimilarities. Principal Coordinate Analysis (PCoA) and Nonmetric multidimensional scaling (NMDS) were utilized to visualize clustering patterns, and statistical significance was tested using Permutational Multivariate Analysis of Variance (PERMANOVA).

**Figure 3:**
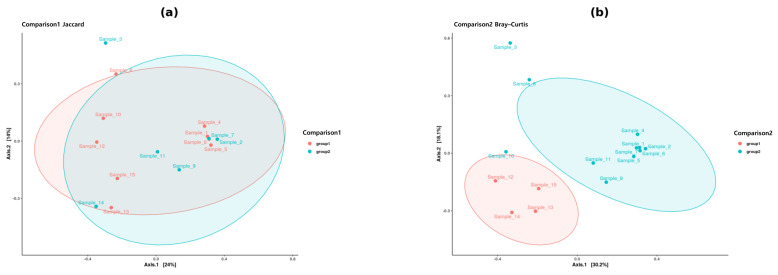
Principal coordinate analysis (PCoA) of beta diversity. (**a**) PCoA plot based on Jaccard distance for Comparison 1 (female vs. male tigers); (**b**) PCoA plot based on Bray–Curtis distance for Comparison 2 (tigers from Uchi Zoo vs. Seoul Grand Park). Ellipses represent 95% confidence intervals for group clustering.

**Figure 4:**
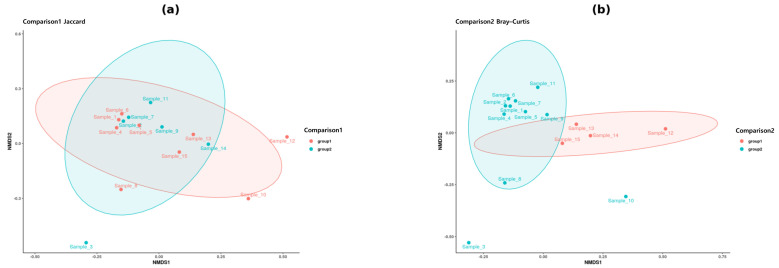
Non-metric multidimensional scaling (NMDS) plots of beta diversity. (**a**) NMDS plot based on Jaccard distance for Comparison 1 (female vs. male tigers); (**b**) NMDS plot based on Bray–Curtis distance for Comparison 2 (tigers from Uchi Zoo vs. Seoul Grand Park). Ellipses represent 95% confidence intervals for group clustering.

In the comparison between female and male tigers (Comparison 1), PCoA plots of Bray–Curtis and Weighted UniFrac distances (Figure 3) illustrated distinct clustering, suggesting compositional differences in the gut microbiome between the sexes. NMDS analysis (Figure 4) further supported this observation, revealing a visible separation in microbial communities between the two groups. However, statistical validation through PERMANOVA (Table 1) revealed no statistically significant differences (*p* > 0.05), suggesting that sex-based variations did not strongly influence gut-microbiome diversity in tigers.

Conversely, the comparison of tigers from Gwangju and Seoul (Comparison 2) revealed distinct clustering based on geographic location in the PCoA plots of Bray–Curtis and Weighted UniFrac distances (Figure 3), suggesting that tigers from these two regions have different gut microbial compositions. NMDS visualization (Figure 4) confirmed this trend, revealing distinct clusters corresponding to each region. PERMANOVA analysis (Table 1) indicated statistically significant differences (*p* < 0.05) in microbial community composition between the two regions. While this suggests that geographical factors may influence gut microbiota in tigers, these findings should be interpreted cautiously given the small sample size and potential confounding with subspecies.

Overall, beta diversity analysis revealed significant compositional differences between tigers from Seoul and Gwangju (Comparison 2; *p* < 0.05), whereas sex-based differences (Comparison 1) were not statistically significant (*p* > 0.05).

### 2.3. Taxonomic Composition

The gut-microbiome composition of 15 tigers was analyzed across five hierarchical taxonomic levels: phylum, class, order, family, and genus. Microbial communities exhibited significant variation among individuals, with distinct patterns observed at each taxonomic level.

**Figure 5:**
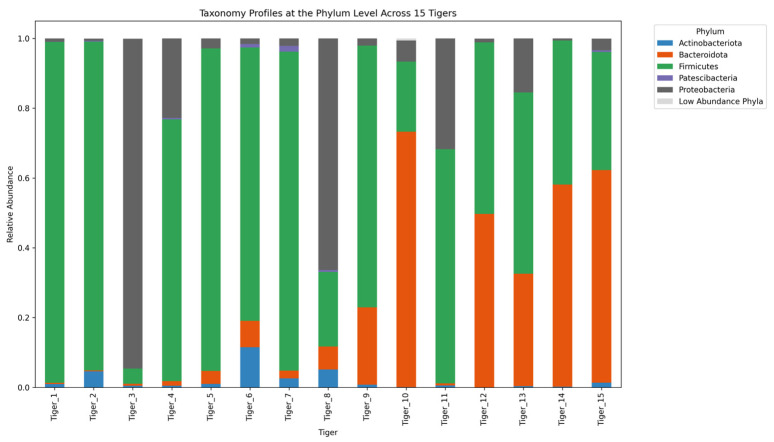
Stacked bar plot illustrating the relative abundance of gut microbiota at the phylum level across 15 tigers.

At the phylum level, the gut microbiota primarily consisted of Firmicutes, Proteobacteria, Bacteroidota, and Actinobacteriota (Figure 5). Firmicutes was the dominant phylum, with relative abundances ranging from 20.08% to 97.67%. Proteobacteria exhibited significant variation, reaching up to 94.47% in Tiger 3 while ranging from 0.55% to 22.77% in most individuals. Bacteroidota was most abundant in Tigers 10 (73.19%) and 15 (60.93%) but remained < 10% in other individuals. Minor phyla, including Fusobacteriota, Cyanobacteria, Verrucomicrobiota, and WPS-2, were detected at lower levels.

**Figure 6:**
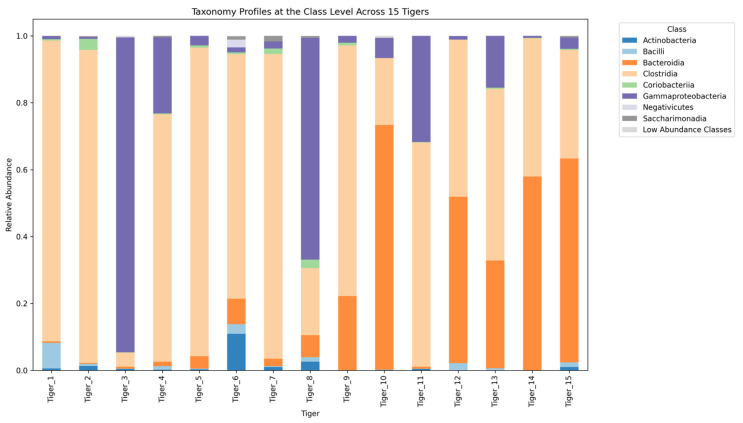
Stacked bar plot illustrating the relative abundance of gut microbiota at the class level across 15 tigers.

At the class level, Clostridia (Firmicutes) was the most prevalent, followed by Gammaproteobacteria, Bacteroidia, and Bacilli (Figure 6). Clostridia was the dominant class, reaching up to 93.59% in Tiger 2 but only 4.24% in Tiger 3. Gammaproteobacteria, which was the most abundant class in Tiger 3 (94.14%), occurred at significantly lower levels in all other individuals. Bacteroidia and Bacilli were moderate across individuals, while Fusobacteriia and Deinococci were detected at minimal levels.

**Figure 7:**
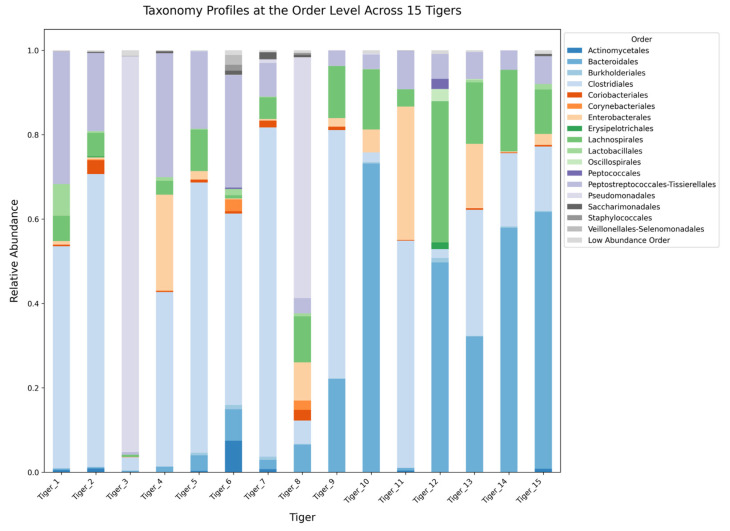
Stacked bar plot illustrating the relative abundance of gut microbiota at the order level across 15 tigers.

Microbial classification at the order level revealed that Clostridiales was the dominant order, particularly in Tiger 2 (69.41%) (Figure 7). Peptostreptococcales-Tissierellales were prevalent in Tigers 1 (31.35%) and 4 (29.34%). Bacteroidales exhibited significant variation, reaching 73.16% in Tiger 10 while remaining < 1% in some individuals. Other significant orders, including Enterobacterales, Lactobacillales, and Lachnospirales, demonstrated varying abundances across tigers.

**Figure 8:**
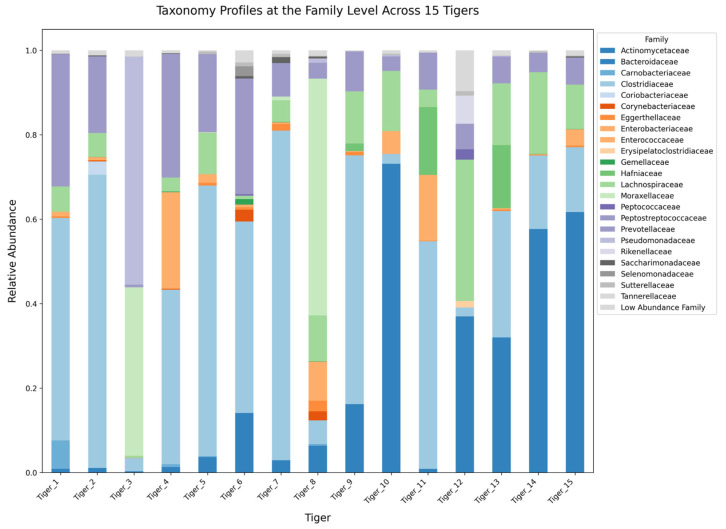
Stacked bar plot illustrating the relative abundance of gut microbiota at the family level across 15 tigers.

At the family level, Lachnospiraceae and Peptostreptococcaceae were the predominant taxa (Figure 8). Lachnospiraceae exhibited high abundance in Tiger 12 (33.48%), while Peptostreptococcaceae dominated across multiple individuals. Bacteroidaceae, a key family within Bacteroidales, was highly enriched in Tiger 10. Enterobacteriaceae, a family belonging to Gammaproteobacteria, demonstrated significant prevalence in Tigers 3 and 8 while remaining much less abundant in other individuals. Minor families, including Erysipelotrichaceae, Fusobacteriaceae, and Corynebacteriaceae, were detected in low proportions.

**Figure 9:**
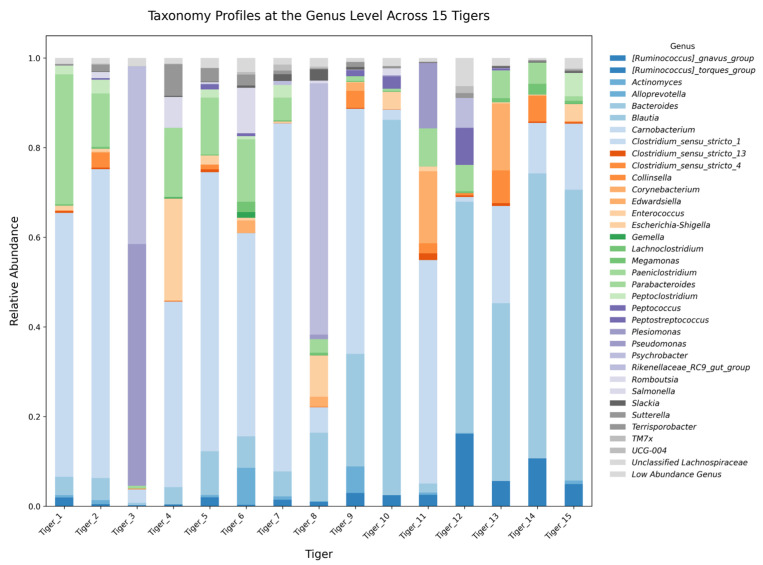
Stacked bar plot illustrating the relative abundance of gut microbiota at the genus level across 15 tigers.

At the genus level, several microbial genera were observed, with Clostridium, Bacteroides, Escherichia, and Lactobacillus being the most frequently detected taxa (Figure 9). Clostridium was the predominant genus in most individuals. Bacteroides exhibited significant abundance in Tiger 10 (73.16%) while remaining at much lower levels in other tigers. Escherichia was the predominant genus in Tiger 3, indicating the high abundance of Gammaproteobacteria in this individual. Lactobacillus exhibited greater abundance in Tigers 5 and 6, while Fusobacterium was detected at relatively low levels across all of the samples.

## 3. Discussion

The gut microbiome is essential for host metabolism, immune function, and overall health, with microbial diversity influenced by host genetics, diet, environmental conditions, and disease status. This study provides a comprehensive comparative analysis of gut microbial diversity across 15 captive tigers, including Siberian and Bengal tigers, housed at two locations in Korea. The findings indicate that gut microbial composition differed between tigers housed in different regions. However, because all Bengal tigers were from one location (Gwangju) and all but one Siberian tiger were from another (Seoul), subspecies and regional effects could not be separated. Therefore, no conclusion can be drawn about the independent effect of subspecies. Moreover, specific taxa were enriched in individuals with underlying health conditions, suggesting a potential association between gut microbiota and disease susceptibility, particularly in tigers with underlying health conditions such as hypothyroidism and chronic renal failure.

Alpha diversity analysis revealed that microbial richness and evenness varied among individuals. Tigers 6 and 2 exhibited the highest richness as measured using Chao1 and Observed Features metrics, while Tigers 1 and 14 demonstrated the lowest richness. In contrast, the Shannon and Simpson indices indicated that Tigers 12, 13, and 14 exhibited the highest evenness, while Tiger 10 demonstrated the lowest. These findings are consistent with those of previous studies on captive tigers, which reported inter-individual variability influenced by environmental and dietary factors [13]. While no significant sex-based differences were observed, microbial evenness differed significantly between geographic groups. Tigers from Seoul Grand Park exhibited greater microbial evenness than those from Uchi Zoo, suggesting that environmental factors, dietary variations, or regional microbial exposures may contribute to microbiome differences [14]. Similar findings have been reported in captive and wild carnivores, where regional variations influence microbiome composition [9].

Beta diversity analysis further confirmed the significant effect of geographic origin on gut microbial composition. PCoA and NMDS based on Bray–Curtis and Weighted UniFrac distances revealed distinct clustering patterns differentiating tigers from Seoul and Gwangju. PERMANOVA confirmed these differences (*p* < 0.05), highlighting the role of regional environmental factors in shaping the microbiome. These findings suggest that geographic location significantly influences gut microbiota composition in captive tigers. Similar trends have been observed in Malayan tigers, where environmental differences across facilities affected microbial diversity [15]. These findings are consistent with those of previous studies on wild Bengal tigers, where distinct microbiome signatures were observed across different habitats [14]. Similarly, studies on wild and captive Siberian tigers in China and Russia demonstrate that captive individuals generally exhibit altered microbiomes characterized by reduced microbial diversity, potentially owing to dietary constraints and controlled feeding regimens [9]. This study supports this hypothesis, as captive tigers from different locations exhibited significant microbial differences despite being housed in controlled environments.

At the phylum level, Firmicutes was the most abundant, followed by Proteobacteria, Bacteroidota, and Actinobacteriota. The high abundance of Firmicutes is consistent with that of previous studies on captive tigers and wild felids, indicating an adaptation of the gut microbiota to a carnivorous diet [13]. However, the overwhelming dominance of Proteobacteria in Tiger 3 (94.47%) suggests potential dysbiosis, as this phylum has been associated with gut inflammation and metabolic disorders [1]. Although elevated Bacteroidota levels were observed in Tigers 10 and 15, it is unlikely that these differences were driven by diet, given that all individuals were fed a nearly identical regimen. Other factors, such as age or disease status, may have contributed, but this could not be statistically verified due to limited sample size. Bacteroidota, including genera such as Bacteroides, are involved in the degradation of complex polysaccharides and production of SCFAs, functions that are generally beneficial for host gut health [9]. However, due to the absence of detailed dietary metadata, functional interpretation remains speculative.

At the class level, Clostridia was the most prevalent, followed by Gammaproteobacteria, Bacteroidia, and Bacilli. The dominance of Clostridia, particularly in Tiger 2 (93.59%), is consistent with its established role in fermenting dietary fibers and producing butyrate, an SCFA essential for colonic health. The high abundance of Gammaproteobacteria in Tiger 3 (94.14%) is consistent with the elevated levels of Proteobacteria at the phylum level, further suggesting potential dysbiosis. Similar patterns have been reported in captive big cats with gastrointestinal disturbances [16]. Bacteroidia, primarily represented by the genus Bacteroides, were significantly abundant in Tigers 10 and 15. Although these taxa have been linked to fiber-rich diets in other species, all of the tigers in this study were fed similar diets, and no differences in fiber intake were documented. Bacilli, which include lactic acid bacteria, were present at moderate levels across individuals, indicating a diverse microbial community [9].

At the order level, Clostridiales was the most abundant, particularly in individuals with stable microbiomes, while Bacteroidales exhibited significant variation, with its highest abundance in Tiger 10 (73.16%). The presence of Enterobacterales in Tiger 3 is of particular concern, as this order has been associated with gut inflammation in captive carnivores [16].

Family-level analysis revealed that Lachnospiraceae and Peptostreptococcaceae were the dominant families across individuals, consistent with their established roles in gut health and SCFA production. However, the overrepresentation of Enterobacteriaceae in Tiger 3 raises concerns about potential microbial dysbiosis, as this bacterial family is often associated with metabolic disorders and gut inflammation in carnivores [16].

At the genus level, *Clostridium*, *Bacteroides*, and *Escherichia* were the most frequently detected taxa. *Clostridium* was predominantly abundant, while *Bacteroides* was significantly elevated in Tiger 10, further supporting the role of diet on microbiome composition. The overrepresentation of *Escherichia* in Tiger 3 provides additional evidence of gut microbial imbalance, as this genus is often associated with inflammatory conditions in mammals [16].

The presence of underlying health conditions influenced the microbiome composition in certain individuals. Tiger 3, diagnosed with hypothyroidism, exhibited a high Proteobacteria and Enterobacteriaceae dominance, consistent with the dysbiosis patterns observed in metabolic disorders [16]. Tiger 10, diagnosed with chronic renal failure, demonstrated lower microbial evenness, consistent with the findings of previous studies on aged and diseased animals [9]. Tiger 12 (mammary tumor) exhibited an altered microbial community, which may influence immune responses. These findings emphasize the importance of monitoring the gut microbiome in captive-tiger populations for health assessments and conservation planning.

Despite these findings, this study has several limitations. The small sample size and uneven distribution of health conditions and age limited our ability to draw robust conclusions about their effects on gut microbiota. Future studies should aim to compare microbial profiles across age-defined groups (e.g., juveniles vs. seniors) and between healthy and diseased individuals to better understand the impact of these factors. While all tigers were born and raised in captivity and the duration of captivity could be inferred from available records, detailed metadata on clinical treatments, antibiotic usage, probiotic administration, and husbandry practices were unavailable. This lack of individual-level information—such as enrichment practices, stress exposure, and animal handling—reduces the interpretive depth of our findings. Enrichment-driven shifts in gut microbiota have also been observed in captive Malayan tigers, highlighting the importance of behavioral and environmental management in shaping microbial communities [17]. In addition, some individuals were littermates (e.g., Tigers #2 and #6, #4 and #5, and #8, #9, and #11), which may have introduced kinship-related microbial similarities due to shared genetics and early-life environments. The potential influence of kinship and early-life exposures should also be accounted for in future study designs. The absence of nutritional metadata, including fiber intake or feeding variation, also restricted our ability to interpret dietary effects. Notably, the wide age range among individuals (1 to 18 years) may have confounded the observed absence of sex-based differences; since gut microbiota varies with age, age-matched comparisons are needed to better understand sex-related patterns. The confounding between subspecies and geographic location further limits statistical power for group comparisons, meaning some observed differences may reflect random variation or unmeasured factors. Lastly, the lack of a comparative wild-tiger group restricts the ability to contextualize our findings within the species’ natural microbiome profile. The inclusion of wild counterparts will be essential for distinguishing captivity-induced microbial changes from species-intrinsic features. Future research incorporating more comprehensive metadata and larger, more genetically diverse cohorts—including wild individuals—will be essential to disentangle these confounding variables. Given the exploratory nature and the small, unbalanced sample size, the findings from this study should be interpreted as preliminary and not generalized beyond the studied cohort.

While preliminary in nature, these findings suggest that geographic origin may shape gut microbial diversity in captive tigers, whereas sex-based differences appear minimal. This exploratory study underscores the potential value of gut-microbiome profiling for health assessments and conservation planning in zoological settings. However, given the confounding between subspecies and housing location, and the small, unbalanced sample size, the results should be interpreted with caution and considered hypothesis-generating. Future studies should aim to validate these trends with larger, balanced cohorts and disentangle the effects of region, subspecies, age, and health status on gut microbiota in captive felids. Microbiome-management strategies should be informed by regional and environmental contexts rather than taxonomic classification. This interpretation aligns with previous findings in other captive big cats, including the seasonal and housing effects observed in captive South China tigers [18].

## 4. Materials and Methods

### 4.1. Ethical Approval

All of the experimental procedures were approved by the Institutional Animal Care and Use Committee at Kyungpook National University (KNU 2024-0026, approval date: 22 January 2024). Fecal samples were collected noninvasively to minimize stress and ensure adherence to ethical research standards for wildlife.

### 4.2. Sample Collection and Health Status

In total, 15 tiger fecal samples were collected from Seoul Grand Park in Seoul (*n* = 11, all Siberian tigers) and Uchi Zoo in Gwangju (*n* = 4, including three Bengal tigers and one Siberian tiger) between November and December 2023. The age, sex, diet, and health conditions of each tiger were recorded (Table 2). At the time of sampling, all individuals were clinically assessed, with some having pre-existing health conditions such as thyroid dysfunction (Tiger 3), chronic renal failure (Tiger 10), and a mammary tumor (Tiger 12). The fecal samples were collected immediately post-defecation using sterile equipment and they were stored in sterile containers. The samples were transported on ice to the laboratory within 24 h and then stored at −80 °C until DNA extraction.

### 4.3. Experimental Grouping for Microbiome Analysis

The tigers were categorized into two comparison groups for microbiome analysis: Comparison 1 (Female vs. Male tigers) and Comparison 2 (Tigers from Gwangju Uchi Zoo vs. Tigers from Seoul Grand Park), based on sex and geographic location (Table 3).

### 4.4. DNA Extraction and 16S rRNA Gene Sequencing

Genomic DNA was extracted from 200 mg of fecal material using the QIAGEN PowerSoil DNA Kit (Qiagen, Hilden, Germany) following the protocol of the manufacturer. DNA concentration and purity were measured using a Nanodrop spectrophotometer (Thermo Fisher Scientific, Waltham, MA, USA) and Qubit fluorometer (Thermo Fisher Scientific, USA). The V3–V4 region of the 16S rRNA gene was amplified using the universal primer pair 16S-F (5′-CCTACGGGNGGCWGCAG-3′) and 16S-R (5′-GACTACHVGGGTATCTAATCC-3′). PCR amplification was performed as previously described [16].

### 4.5. Library Preparation and Sequencing

The PCR products were purified using AMPure XP beads (Beckman Coulter, Brea, CA, USA) to eliminate residual primers and contaminants. Purified amplicons were quantified using a Qubit fluorometer and were pooled in equimolar concentrations to construct the sequencing library. The library was prepared using the Nextera XT DNA Library Preparation Kit (Illumina, San Diego, CA, USA) according to the protocol of the manufacturer. Sequencing was performed on an Illumina MiSeq platform using 2 × 300 bp paired-end reads. Quality control and cluster generation were automated, ensuring a uniform distribution of sequence reads across the samples.

### 4.6. Quality Control and Preprocessing

Raw sequencing reads were assessed using FastQC (v0.10.1, Babraham Institute, Cambridge, UK) to evaluate base quality, GC content, and the presence of adapter sequences. To ensure high-quality data, reads with a Phred score < 20 were trimmed using Cutadapt (v3.2, Marcel Martin, Freiburg, Germany), effectively removing low-quality bases and any residual adapter sequences. Subsequently, the DADA2 pipeline (v1.20.0, R Development Core Team, Vienna, Austria) [19] was employed for further quality filtering, denoising, and chimera removal, generating amplicon sequence variants (ASVs) with enhanced taxonomic resolution compared to that of traditional operational taxonomic units (OTUs). The DADA2 parameters were set as follows: maxEE = 2 (maximum expected errors), truncQ = 2 (quality score threshold for truncation), truncLen = c (250, 200) (truncation lengths for forward and reverse reads), and maxN = 0 (no ambiguous bases allowed). After preprocessing, sequences were demultiplexed before being analyzed using the QIIME2 pipeline (v2021.11, Caporaso Lab, Flagstaff, AZ, USA) for taxonomic classification [20]. ASVs were taxonomically assigned using the SILVA reference database (v138.99, Max Planck Institute, Bremen, Germany). For comparison, OTUs were clustered at a 97% similarity threshold using the Greengenes reference database (v13_5, Lawrence Berkeley National Laboratory, Berkeley, CA, USA) [21].

### 4.7. Alpha Diversity Analysis

Alpha diversity indices were calculated to assess microbial richness, evenness, and phylogenetic diversity. The following indices were included: Chao1 Index and Observed Features (Species richness), Shannon Entropy and Simpson Index (Overall diversity) Simpson Evenness and Pielou’s Evenness (Species evenness), and Faith PD (Phylogenetic richness). Statistical comparisons between experimental groups were conducted using Wilcoxon Rank-Sum Tests with a significance threshold of *p* < 0.05.

### 4.8. Beta Diversity Analysis

Beta diversity was assessed using the following distance metrics: Jaccard (qualitative measure of community dissimilarity), Bray–Curtis (quantitative measure incorporating relative abundance), Unweighted UniFrac (qualitative metric considering phylogenetic relationships), and Weighted UniFrac Distance (quantitative metric incorporating phylogenetic relationships).

PCoA was performed on the Bray–Curtis dissimilarity data to visualize microbial community separation based on their compositional differences. NMDS was employed for the Jaccard distance, unweighted UniFrac, and weighted UniFrac metrics, providing complementary insights into the microbial community differences.

To statistically assess the differences in beta diversity between the tiger groups, PERMANOVA was conducted using the adonis2 function within the vegan R package (v2.5–7, R Core Team, Vienna, Austria). This analysis was conducted using 999 permutations to evaluate the significance of the group differences [22].

### 4.9. Taxonomic Composition Analysis

The taxonomic composition of the microbial communities was analyzed at the phylum, class, order, family, and genus levels using the SILVA database (v138.99, Max Planck Institute, Bremen, Germany) for taxonomic classification. Low-abundance taxa (<1% across all samples) were grouped as “Low Abundance Taxa.” Taxonomic visualization was performed using stacked bar plots. Differential taxon abundance was evaluated using the Analysis of Composition of Microbiomes with Bias Correction to account for compositional bias [23].

## 5. Conclusions

This exploratory study offers preliminary insights into the gut microbiome composition of 15 captive tigers, including Siberian and Bengal subspecies, housed in two different regions of Korea. Based on 16S rRNA gene sequencing of fecal samples, we observed that geographic origin may influence microbial community structure, while sex-based differences appeared minimal. These findings suggest that environmental factors, including regional husbandry practices and zoo conditions, could play a role in shaping gut microbiota in captive felids. However, due to the small sample size and confounding between subspecies and geographic location, the results should be interpreted with caution and cannot be generalized beyond the studied cohort. The uneven distribution of age and health status also limited statistical assessment of these variables. Future studies incorporating metagenomic and metabolomic approaches, as well as age-matched and health-balanced cohorts, will be necessary to elucidate the functional implications of microbial variation and to identify potential biomarkers for health monitoring. Overall, this study highlights the value of microbiome research as a tool for informing conservation and veterinary-management strategies in captive endangered species.

## Figures and Tables

**Table 1:** PERMANOVA results for beta diversity comparisons using Bray–Curtis and Weighted UniFrac distances.

Comparison	Distance Metric	PERMANOVA *p*-Value	Significance
Comparison 1 (Female vs. Male)	Bray–Curtis	>0.05	Not significant
Comparison 1 (Female vs. Male)	Weighted UniFrac	>0.05	Not significant
Comparison 2 (Gwangju vs. Seoul)	Bray–Curtis	<0.05	Significant
Comparison 2 (Gwangju vs. Seoul)	Weighted UniFrac	<0.05	Significant

**Table 2:** Metadata of 15 captive tigers used in microbiome analysis, including sample location, subspecies, sex, diet, and health status at the time of fecal collection.

Tiger ID	Sample Location	Subspecies	Name	Sample Collection Date	Birth Year	Sex	Diet	Health Status
1	Seoul Grand Park	Siberian Tiger	Penza	November 2023	2010	Female	Chicken: 3–5 kg, Beef: 2–3 kg, Rabbit (once a week)	Healthy
2	Seoul Grand Park	Siberian Tiger	Seonho	November 2023	2013	Male	Chicken: 3–5 kg, Beef: 2–3 kg, Rabbit (once a week)	Born together with #6
3	Seoul Grand Park	Siberian Tiger	Rostov	November 2023	2010	Male	Chicken: 3–5 kg, Beef: 2–3 kg, Rabbit (once a week)	Hypothyroidism
4	Seoul Grand Park	Siberian Tiger	Sarang	November 2023	2022	Female	Chicken: 3–5 kg, Beef: 2–3 kg, Rabbit (once a week)	Born together with #5
5	Seoul Grand Park	Siberian Tiger	Haerang	November 2023	2022	Female	Chicken: 3–5 kg, Beef: 2–3 kg, Rabbit (once a week)	Born together with #4
6	Seoul Grand Park	Siberian Tiger	Miho	November 2023	2013	Female	Chicken: 3–5 kg, Beef: 2–3 kg, Rabbit (once a week)	Born together with #2
7	Seoul Grand Park	Siberian Tiger	Joseph	November 2023	2011	Male	Chicken: 3–5 kg, Beef: 2–3 kg, Rabbit (once a week)	Healthy
8	Seoul Grand Park	Siberian Tiger	Geumgang	November 2023	2018	Female	Chicken: 3–5 kg, Beef: 2–3 kg, Rabbit (once a week)	Born together with #9, #11
9	Seoul Grand Park	Siberian Tiger	Baekdu	December 2023	2018	Male	Chicken: (3–5 kg, Beef: 2–3 kg	Healthy
10	Seoul Grand Park	Siberian Tiger	Beautiful	December 2023	2005	Female	Chicken: 3–5 kg, Beef: 2–3 kg, Rabbit (once a week)	Chronic renal failure
11	Seoul Grand Park	Siberian Tiger	Taebaek	December 2023	2018	Male	Chicken: 3–5 kg, Beef: 2–3 kg, Rabbit (once a week)	Born together with #8, #9
12	Uchi Zoo	Bengal Tiger	Love	December 2023	2009	Female	Chicken: 3–4.5 kg	Mammary tumor
13	Uchi Zoo	Bengal Tiger	Ho Soon	December 2023	2012	Female	Chicken: 3–4.5 kg	Healthy
14	Uchi Zoo	Bengal Tiger	Parker	December 2023	2011	Male	Chicken: 3–4.5 kg	Healthy
15	Uchi Zoo	Siberian Tiger	Homin	December 2023	2007	Female	Chicken: 3–4.5 kg	Healthy

**Table 3:** Grouping criteria for comparative microbiome analysis. Comparison 1: sex-based grouping (female vs. male); Comparison 2: region-based grouping (Uchi Zoo vs. Seoul Grand Park).

Comparison	Group A (*n* = )	Group B (*n* = )	Criteria
Comparison 1	Samples 1, 4, 5, 6, 8, 10, 12, 13, and 15	Samples 2, 3, 7, 9, 11, and 14	Female vs. Male
Comparison 2	Samples 12, 13, 14, and 15	Samples 1, 2, 3, 4, 5, 6, 7, 8, 9, 10, and 11	Gwangju (Uchi Zoo) vs. Seoul Grand Park

## Data Availability

The data presented in this study are available on request from the corresponding author. The data are not publicly available due to privacy concerns regarding the individual tigers involved in the study conducted at the Korean Tiger Conservation Center (Baekdudaegan National Arboretum), Conservation and Health Center (Seoul Zoo), and by the Veterinary Medicine Team (Uchi Park Zoo).

## Abbreviations

The following abbreviations are used in this manuscript:

SCFA	short-chain fatty acids
Faith PD	Faith’s Phylogenetic Diversity
PCoA	Principal Coordinate Analysis
NMDS	Nonmetric multidimensional scaling
PERMANOVA	Permutational Multivariate Analysis of Variance
